# High-sensitivity HLA typing by Saturated Tiling Capture Sequencing (STC-Seq)

**DOI:** 10.1186/s12864-018-4431-5

**Published:** 2018-01-15

**Authors:** Yang Jiao, Ran Li, Chao Wu, Yibin Ding, Yanning Liu, Danmei Jia, Lifeng Wang, Xiang Xu, Jing Zhu, Min Zheng, Junling Jia

**Affiliations:** 1 Life Sciences Institute and Innovation Center for Cell Signaling Network, Zhejiang University, Hangzhou Zhejiang, 310058 People’s Republic of China; 2 Key Laboratory of Precision Diagnosis and Treatment for Hepatobiliary and Pancreatic Tumor of Zhejiang Province, First Affiliated Hospital, Zhejiang University School of Medicine, Hangzhou, China; 3 Collaborative Innovation Center for Diagnosis and Treatment of Infectious Diseases, Zhejiang University, Hangzhou, Zhejiang 310003 People’s Republic of China; 4 State Key Laboratory for Diagnosis and Treatment of Infectious Diseases, The First Affiliated Hospital, Zhejiang University, Hangzhou, Zhejiang 310003 People’s Republic of China; 5 School of Mathematical Science, Zhejiang University, Hangzhou, Zhejiang 310058 People’s Republic of China; 6 Beijing Ming-tian Genetics Ltd, Beijing, 100070 People’s Republic of China

**Keywords:** Human leukocyte antigen (HLA), Hybridization capture, HLA typing, Next-generation sequencing (NGS), Third-generation sequencing

## Abstract

**Background:**

Highly polymorphic human leukocyte antigen (HLA) genes are responsible for fine-tuning the adaptive immune system. High-resolution HLA typing is important for the treatment of autoimmune and infectious diseases. Additionally, it is routinely performed for identifying matched donors in transplantation medicine. Although many HLA typing approaches have been developed, the complexity, low-efficiency and high-cost of current HLA-typing assays limit their application in population-based high-throughput HLA typing for donors, which is required for creating large-scale databases for transplantation and precision medicine.

**Results:**

Here, we present a cost-efficient Saturated Tiling Capture Sequencing (STC-Seq) approach to capturing 14 HLA class I and II genes. The highly efficient capture (an approximately 23,000-fold enrichment) of these genes allows for simplified allele calling. Tests on five genes (HLA-A/B/C/DRB1/DQB1) from 31 human samples and 351 datasets using STC-Seq showed results that were 98% consistent with the known two sets of digitals (field1 and field2) genotypes. Additionally, STC can capture genomic DNA fragments longer than 3 kb from HLA loci, making the library compatible with the third-generation sequencing.

**Conclusions:**

STC-Seq is a highly accurate and cost-efficient method for HLA typing which can be used to facilitate the establishment of population-based HLA databases for the precision and transplantation medicine.

**Electronic supplementary material:**

The online version of this article (10.1186/s12864-018-4431-5) contains supplementary material, which is available to authorized users.

## Background

The human leukocyte antigen (HLA) complex is located on chromosome 6p21 which encodes major histocompatibility complex (MHC) proteins involved in immune functions [1, 2]. The highly polymorphic HLA class I (A, B and C) and II (DRB1 and DQB1) genes are crucial in immune rejection of transplantations, immune response to infections, pathogenesis of autoimmune diseases, adverse reactions to medications and cancer development [3–5]. Thus, identifying HLA polymorphisms, also called HLA typing, is clinically important.

According to the IMGT/HLA database [6], there are over 3600 alleles for HLA-A, 4400 alleles for HLA-B, 3200 alleles for HLA-C and 1900 alleles for HLA-DRB1. Additionally, the coding sequences of HLA genes of the same class are highly homologous. Many methods for high-resolution typing (two-field resolution, protein-coding variant) of HLA-A/B/C/DRB1/DQB1 have been successfully established, such as sequence-specific oligonucleotide probes (SSOP), sequence-specific primers (SSP) and Sanger-sequencing-based typing (SBT) [7–13]. For high-resolution typing (the first and second field), these methods involve iterative procedures that start with low-resolution typing followed by additional characterizations. Consequently, these methods are both time and labor intensive prohibiting them for high-throughput processing. In addition, there are shortcomings with Sanger sequencing-based approaches because variants in an amplicon cannot be phased leading to cis/trans ambiguities [14, 15].

Next-generation sequencing (NGS) technology developed in the last decade has been widely used in medicine [16]. NGS has advantages of providing single DNA molecule sequence data in a high-throughput manner which allows for highly confident HLA allele determination [17, 18]. NGS-based HLA typing methods rely on either amplicon-based or hybridization-based enrichment of HLA loci followed by massively parallel sequencing [19]. Amplicon-based capture is laborious and requires extensive PCR optimization; hybridization-based enrichment requires an expensive, high-quality probe-pool to cover all of the allelic variations of the targeted HLA genes. These drawbacks make it difficult for large-scale HLA typing.

Here, we used low-cost on-chip long HLA cDNA fragments as baits to capture the coding regions of 14 HLA class I and II genes (HLA-A/B/C/DPA1/DPB1/DQA1/DQB1/DRA/DRB1/DRB3/DRB4/DRB5/E/G). The use of high-density on-chip baits allowed us to capture the coding regions of the HLA genes with very high coverage. This advantage improves the accuracy of HLA typing for five genes (HLA-A/B/C/DRB1/DQB1) with a novel high-performance analysis pipeline compared to a previously reported hybridization-based NGS HLA typing approach [20].

## Results

**Fig. 1:**
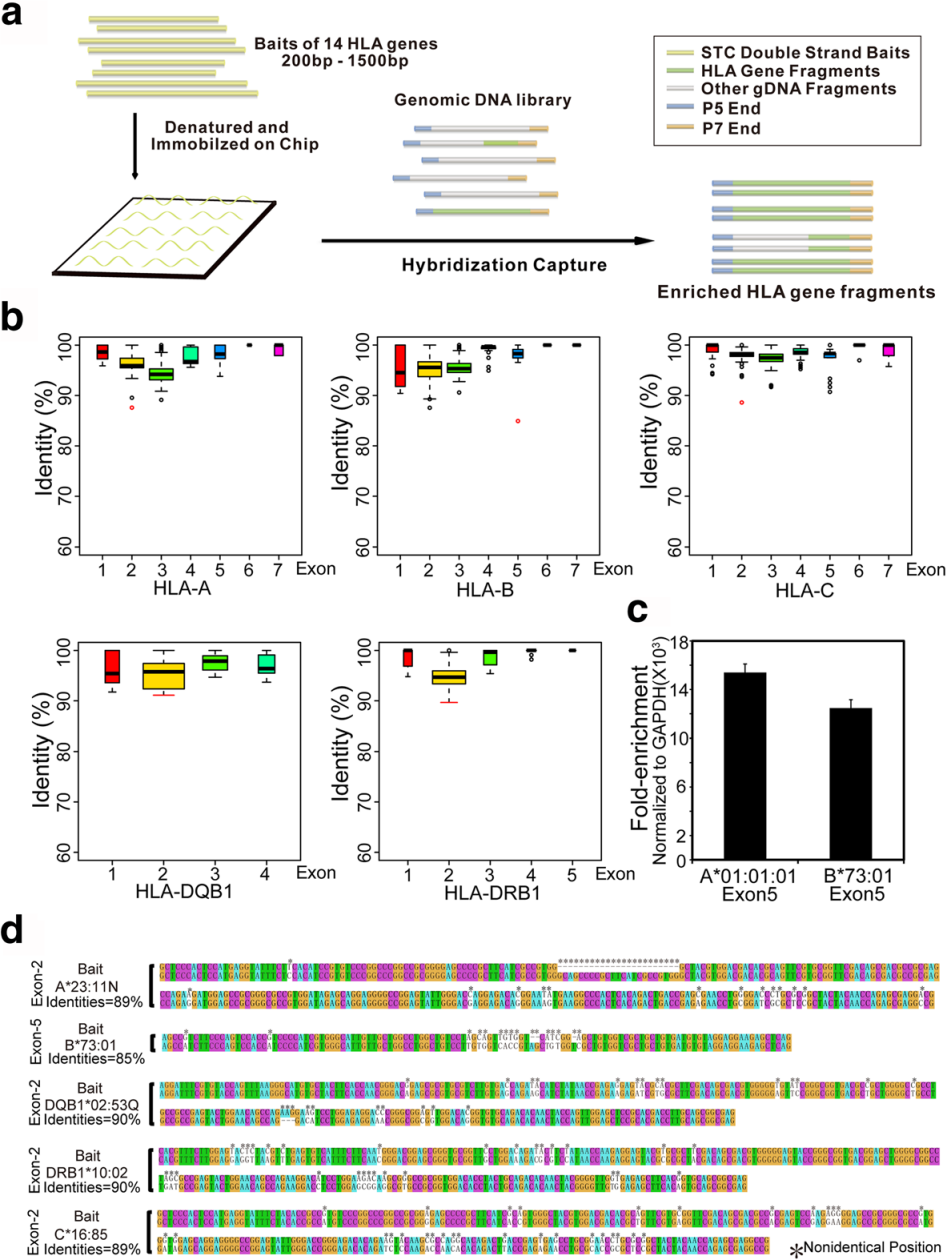
Saturated Tiling Capture Sequencing (STC-Seq) can efficiently capture the exons (> 23 bp) of all IMGT documented HLA-A/B/C/DQB1/DRB1 alleles. **a** The experimental pipeline of Saturated Tiling Capture Sequencing (STC-Seq). **b** Sequence identity between the STC baits and exons (> 23 bp) of all IMGT documented HLA-A/B/C/DQB1/DRB1 alleles. The sequence identity was evaluated based on the number of matched bases normalized by the exon length. **c** The fold-enrichment of the exon 5 of HLA-A*01:01:01 and HLA-B*73:01 after hybridization capture. The fold-enrichment was measured by real-time PCR. **d** The exons (> 23 bp) of the HLA-A/B/C/DQB1/DRB1 alleles having the minimum sequence identity with the corresponding STC baits

To perform targeted sequencing of the coding regions of 14 HLA genes, we selected the HLA alleles with the longest coding regions from the IMGT/HLA reference database (release 3.26) which were used as the bait panel: HLA-A*01:01:01, B*08:01:01, C*16:01:01, DPA1*02:01:01, DPB1*01:01:01, DQA1*01:03:01, DQB1*03:02:01, DRA*01:01:01, DRB1*13:03:01, DRB3*01:01:02, DRB4*01:03:01, DRB5*01:01:01, E*01:01:01 and G*01:01:02. We first amplified plasmids containing the coding sequences (CDS) of the 14 HLA alleles in bacteria. Then, plasmid DNAs were briefly fragmented, pooled, denatured and immobilized on the surface of a nylon chip. We performed two rounds of hybridization to capture both strands of the coding regions of the 14 HLA genes from an NGS genomic DNA library (Fig. 1a). After fragmentation, the length distribution of the baits was from 200 bp to 1500 bp (Additional file 1: Figure S1), and the number of molecule of each on-chip bait was approximately 4.02 × 10^10^ (Table 1). We compared the variation between the baits and all of the IMGT documented alleles of the same genes. More than 99.6% of the exons (> 23 bp) have over 90% sequence identity and 100% of the exons (> 23 bp) have over 85% identity between the baits and reported alleles (Fig. 1b, Additional file 2: Figure S2). We also used a 120 bp fragment which has only 85% matched base with an on-chip probe to mimic the lowest homologous exon (B*73:01 exon 5) shown in Fig. 1b. As expected, the DNA fragment can be efficiently pulled down by the capture chip (Fig. 1c). Previous work has shown that 20 bp to 150 bp complementary sequences can efficiently support hybridization [21–23]. Next, we checked every base of all IMGT documented HLA alleles of the 14 genes. We found that every base of the exons (>23 bp) of all documented HLA alleles of 14 HLA genes can be covered by at least one DNA fragment that had enough complementary sequence with an on-chip bait to enable hybridization capture (Fig. 1d, Additional file 3: Figure S3).

**Table 1:** Number of molecule of on-chip baits

Gene	Mole	Gene	Mole
HLA-A	4.27E + 10	HLA-DRA	2.39E + 10
HLA-B	4.89E + 10	HLA-DRB1	4.42E + 10
HLA-C	4.97E + 10	HLA-DRB3	4.39E + 10
HLA-DPA1	4.48E + 10	HLA-DRB4	4.39E + 10
HLA-DPB1	4.56E + 10	HLA-DRB5	4.36E + 10
HLA-DQA1	2.34E + 10	HLA-E	3.04E + 10
HLA-DQB1	4.60E + 10	HLA-G	3.28E + 10

**Fig. 2:**
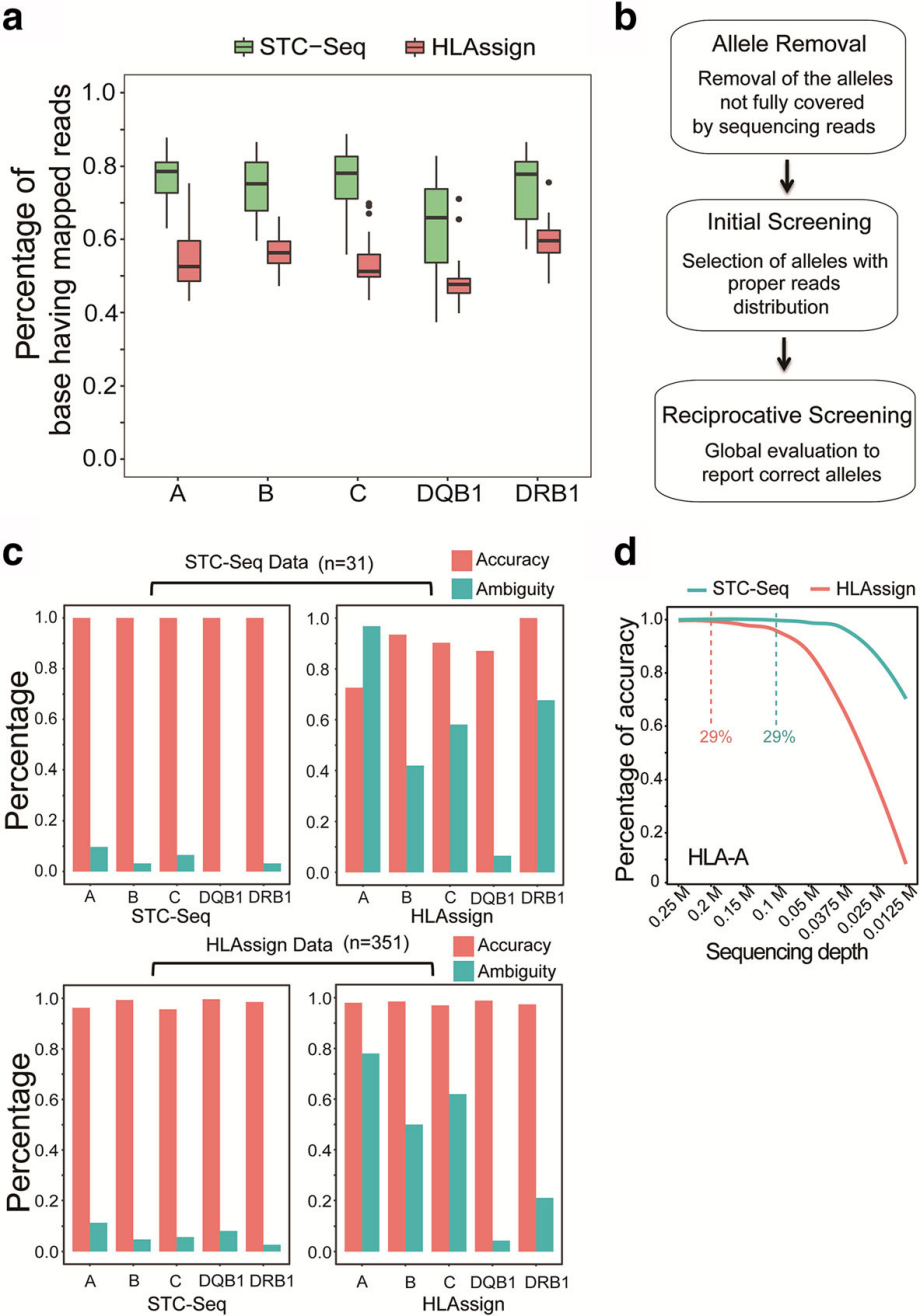
Comparison of HLA-typing accuracy between STC-Seq and HLAssign. **a** Comparison of the percentages of bases having mapped reads between STC-seq and HLAssign. The HLA-A/B/C/DQB1/DRB1 core exons (2,3,4) were compared. Only the positions of the first bases of the mapped reads were considered. **b** Overview of the STC-Seq analysis pipeline. **c** Comparison of typing accuracy of HLA-A/B/C/DQB1/DRB1 between STC-seq and HLAssign. **d** Comparison of the typing accuracy of HLA-A between 31 STC-Seq datasets and 31 HLAssign datasets at different sequencing depths using STC-seq analysis pipeline. The dashed lines indicate the sequencing depths of STC-seq and HLAssign datasets at a threshold with 29% exon bases having mapped reads, below which the typing accuracy drops significantly

We tested the hybridization capture efficiency of STC on 14 HLA genes using 31 samples. After capture, the target regions were enriched, on average, by 23,038-fold and, on average, 73% of bases (range 37.4–87.8%) inside the coding region were covered by mapped reads (only the positions of the first base of the mapped reads were counted) at a 0.25 M sequencing depth (Fig. 2a). By contrast, a previously reported bead-based oligonucleotide capture system only showed a 700-fold enrichment [20] and, on average, 54.5% bases (range 39.8–75.6%) of the coding regions had mapped reads at a similar sequencing depth (Fig. 2a). These data indicate that the extraordinary length (200–1500 bp) and a high number of the on-chip double-stranded baits make it possible to acquire high complexity data for the captured HLA DNA fragments.

The high diversity of mapped reads in the HLA regions provides information about the genetic linkage of polymorphisms. Therefore, it is easy to exclude most alleles with abnormal mapping, which makes the subsequent HLA allele calling more straight-forward. To perform HLA calling, we used the base coverage information and distribution of the first bases of the mapped reads to perform two rounds of screening. We first removed the alleles that did not have complete base coverage inside large exons (> = 70 bp) by a 70 bp continuously aligned region of any sequencing read. Next, we used a window (22 bp by default) to scan the coding region of the remaining alleles and count the number of mapped reads inside each window (only the positions of the first base of the mapped reads were counted). We removed the alleles that contained a window with zero mapped reads, but the corresponding window of any other allele(s) of the same gene had mapped reads. After these two rounds of filtration, in the majority of cases, less than 100 candidate alleles remained for the 14 genes. To further narrow the candidate alleles, we used a genotyping strategy which is based on the hypothesis that the correct genotype could maximally explain the mapped reads on the remaining candidate alleles. In the first step, we randomly paired the remaining alleles of the same gene, counted the relative amounts of unique mapped reads between the two alleles (see details in Methods) and removed the allele(s) that had 15-fold fewer unique mapped reads compared to their partners. Then, we randomly paired the remaining alleles of the same gene and reported the allele pair(s) that maximally explained the mapped reads of remaining alleles of same genes together with the correct alleles of the other HLA genes (Fig. 2b) (for details, see the Methods).

We tested STC-seq analysis pipeline on 382 datasets (31 datasets are generated by STC-Seq, and 351 datasets are from a previously published work [20]) with known allele types for the HLA-A, B, C, DQB1 and DRB1 genes. The results for all five HLA genes were consistent (98% correct, 2% incorrect, 6.3% ambiguity) (Fig. 2c). We also tested a previously reported algorithm, HLAssign, on STC-seq datasets. However, its results were less consistent. (88.7% correct, 11.3% incorrect, 54.2% ambiguity) (Fig. 2c). Because our allele-calling pipeline considered the interference of multi-gene mapped reads, STC-seq reported significantly fewer ambiguous allele combinations than HLAssign on the sequencing data of both STC-Seq and HLAssign [20] (Fig. 2c, Additional file 4: Table S1). We also checked the correlation between the percentage of exon bases having mapped reads and HLA typing accuracy. We found that approximately 29% of exon bases with mapped reads was the threshold below which the typing accuracy dropped dramatically (Fig. 2d, Additional file 4: Table S1). STC-seq requires 0.1 M reads (100 bp single-end) to reach this threshold for typing the 14 HLA genes, whereas the HLAssign needs 0.2 M reads to reach the same threshold (Fig. 2d). In summary, because of improved capture efficiency and the high performance of the analysis pipeline, the HLA typing accuracy of STC-Seq is better than HLAssign, a hybridization-based NGS approach (Fig. 2c, d).

**Fig. 3:**
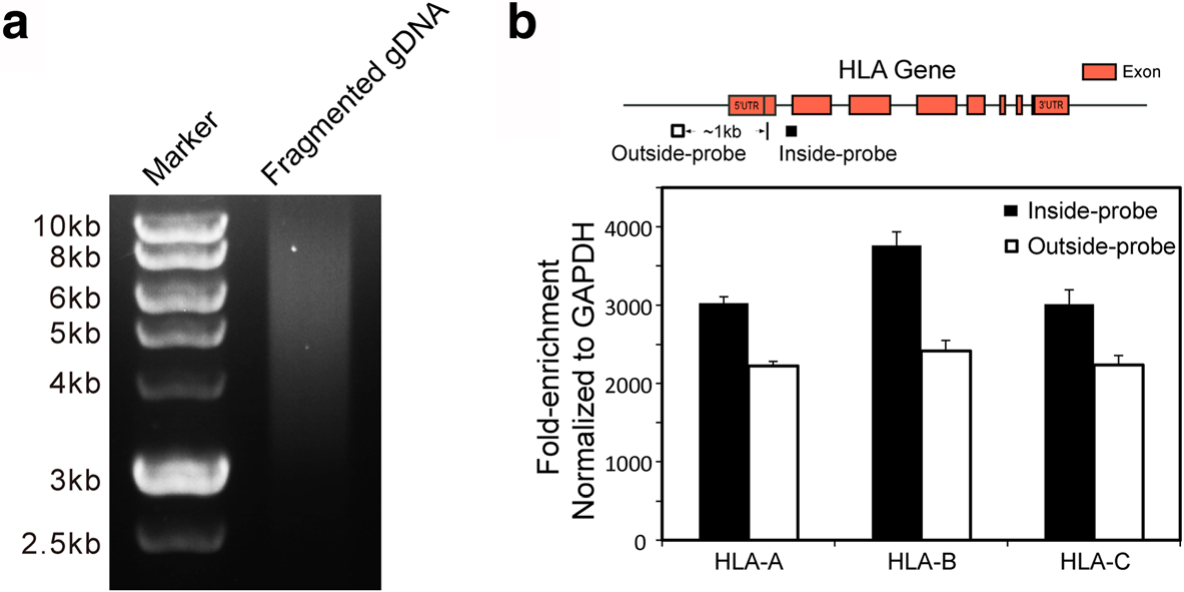
STC can capture integral loci of HLA genes. **a** Gel image showing the size distribution of the input gDNA. **b** The fold-enrichment of the targeted HLA loci after hybridization capture. The fold-enrichment is measured by real-time PCR. Outside and inside primer pairs were used

The lengths of the highly homologous regions between HLA genes can extend beyond the read-length of most NGS platforms. This causes many diploid ambiguities and is a significant drawback of NGS-based HLA typing approaches. Third-generation sequencing platforms, such as PacBio SMRT Sequencing, deliver single-molecule observations with long reads that are capable of spanning the majority of HLA class I and II genes. Direct sequencing of full-length HLA genes can provide directly phased and high-resolution results [24]. Because of the high similarity between STC long baits and the corresponding HLA genes, STC can capture an integral HLA locus for third-generation sequencing-based HLA typing. As a proof of principle, we used STC baits for the HLA-A, B and C genes to capture their targets from a 3–10 kb fragmented human genomic DNA (Fig. 3a). As expected, STC successfully enriched (by more than 2000-fold) the integral loci of the HLA-A, B and C genes (Fig. 3b).

## Discussion

In this work, we developed an NGS-based high-resolution HLA genotyping workflow (STC-Seq) and HLA allele analysis pipeline. Instead of conventional in-solution hybridization capture approaches, STC-Seq uses long double-stranded HLA cDNA fragments as baits. STC-Seq provided an average 23,038-fold enrichment of the coding regions of the 14 HLA genes. We also presented a high-resolution HLA-calling pipeline that reached 98% consistency and 6.3% ambiguity for analyzing the HLA-A/B/C/DRB1/DQB1 loci on STC-seq datasets and previously published NGS datasets [20] (Fig. 2c). This HLA-calling pipeline also outputs high-resolution results for the HLA-DQA1/DRA/DPA1/DRB1/DRB3/DRB4/DRB5/E/G genes captured and sequenced by STC-Seq.

Recently, several NGS-based HLA class I and II typing methods have been reported [25]. They all require enrichment of HLA DNA fragments over the genomic background before sequencing. Although amplicon-based enrichment is a widely used approach [26, 27], the amplicon size is restricted due to the short NGS reads. Tedious primer optimization steps are required to improve coverage and avoid co-amplification of pseudogenes and highly homologous HLA loci [20]. Additionally, amplification bias and target dropouts occur frequently [28, 29]. In this work, we chose a hybridization-based approach that can fit a wide range of target size and has greater flexibility in adding new target genes. Based on the characterization of the probe and capture matrix, there are three documented hybridization-based capture methods: i) bead-based enrichment using biotin-labeled oligonucleotide baits [30, 31]; ii) solid-based enrichment using oligonucleotide baits [32, 33]; and iii) solid-based enrichment method using large gDNA or cDNA fragments [34–36]. The enrichment of targeted HLA regions is moderate using oligonucleotide baits, as reported by a previous work [20], and the cost of beads and in vitro synthesized oligonucleotide baits is very high. To allow high-resolution HLA-typing of large populations at 5–10% cost of current common hybridization-based and amplicon-based NGS approaches (for measurement details, see the Methods), we developed STC-Seq to capture and sequence the coding region of 14 HLA genes using large CDS fragment as baits. Previous work has shown that 20 to 150 bp complementary sequences can efficiently generate hybridization signals [21–23], which explains why the long CDS fragments of one HLA allele could serve as a universal bait to efficiently pull down the corresponding homologous alleles. Moreover, long double-stranded baits can acquire a high diversity of captured reads, which contributes to the improvement of the HLA typing accuracy comparing to a hybridization-based NGS HLA typing approach [20]. Currently, there are nine documented HLA null alleles because of their special intronic sequences. These intronic sequences are all in the 10 bp regions of intron/exon boundaries and can be efficiently pulled down by STC on-chip probes (Additional file 5: Table S2). We also provided a script to ID these null alleles in the analysis pipeline (for details, see Methods).

Current NGS HLA typing approaches are not effective in identifying novel alleles due to their short sequence reads [20]. The read lengths of third-generation single-molecule sequencing can reach 20 kb with high quality [37–39]. Because the genomic loci of class I and II HLA alleles are between approximately 1 kb and 17 kb [40], third-generation single-molecule sequencing can directly provide HLA allele-level resolution and should be the ultimate solution to identify novel HLA alleles [41]. Although PCR amplicon approaches have been successfully tested for HLA-A, -B and -C in third-generation sequencing [41], the potential risk of long-template PCR artifacts (i.e., chimeras, mutations and drop-off) is high [42–44]. We reason that the double-stranded long baits of STC-seq should be a better enrichment approach for third-generation sequencing-based HLA typing.

## Conclusions

In summary, we developed a high-resolution, low-cost and highly accurate HLA typing pipeline, STC-seq, that does not require the expensive reagents of hybridization-based enrichment (i.e., beads and oligonucleotide baits) or laborious steps of amplicon-based enrichment. These advantages of STC-Seq can significantly facilitate the establishment of population-based HLA databases for the precision and transplantation medicine.

## Methods

### Sample

Twenty-six genomic DNA (gDNA) samples with known first two sets of digitals (field1 and field2) genotypes and five genomic DNA (gDNA) samples with known first three sets of digitals genotypes of HLA-A/B/C/DRB1/DQB1 were obtained from the China Marrow Donor Program (CMDP).

### NGS library preparation

Genomic DNA samples were sonicated to an average fragment size of 300 bp. One-hundred nanograms of fragmented gDNA was used for DNA library construction with the NEBNext Ultra II DNA Library Prep Kit (E7645) according to the manufacturer’s protocol.

### Preparation of HLA gene capture chip

Plasmids containing the whole coding sequences (CDS) of 14 HLA genes (HLA-A*01:01:01, B*08:01:01, C*16:01:01, DPA1*02:01:01, DPB1*01:01:01, DQA1*01:03:01, DQB1*03:02:01, DRA*01:01:01, DRB1*13:03:01, DRB3*01:01:02, DRB4*01:03:01, DRB5*01:01:01, E*01:01:01, G*01:01:02) were equally mixed (1 μg of total plasmid DNA). The DNA mixture was briefly fragmented into 200-1500 bp fragments by sonication. These bait fragments were denatured in 0.5 M NaOH at room temperature for 20 min. To prepare the capture chip, denatured baits were applied to a 40 mm^2^ Nylon chip and vacuum dried at 80 °C for 1 h. The capture chip can be stored in dry conditions at room temperature for at least 3 months.

### Hybridization capture of HLA fragments and NGS sequencing

Whole genomic DNA libraries (1 μg each) were mixed with the adaptor blockers and 5 μg of Cot-I DNA. The DNA mixture was denatured at 95 °C for 5 min and then snap cooled on ice immediately. Next, the denatured DNA mixture and capture chip were transferred into hybridization solution (6xSSC, 1% SDS, 5× Denhardt’s Solution). Hybridization was performed at 65 °C for 4 h. After hybridization, the capture chip was washed with 2× SSC and 0.1% SDS for 5 min and 0.2× SSC and 0.1% SDS for 2 × 5 min at 55 °C. The captured DNAs were eluted with 100 μl of TE at 95 °C for 10 min and purified by using a PCR clean-up kit (Qiagen). The eluted DNAs were subjected to 15 cycles of PCR amplification using the Illumina P5 and P7 primers and subjected to another round of hybridization capture with the same conditions.

After enrichment, DNA samples were sequenced on the Illumina HiSeq 2500 platform with the single-end 100 bp model.

### Blocker sequence

P5-blocker: AGATCGGAAGAGCGTCGTGTAGGGAAAGAGTGTdIdIdIdIdIdIdIdIGTGTAGATCTCGGTGGTCGCCGTATCAT-ddC.

P7-blocker: CAAGCAGAAGACGGCATACGAGATdIdIdIdIdIdIdIdIGTGACTGGAGTTCAGACGTGTGCTCTTCCGAT-ddC.

### Capture of integral loci of HLA genes

Genomic DNA was briefly fragmented to produce fragments larger than 3 kb. Five-hundred nanograms of fragmented gDNA was used as the input for capture-enrichment according to the method described above. After elution, RT-PCR was performed.

### Capture of B*73:01 exon5

The sequence of B*73:01 exon5 was synthesized by Synbio Biotechnologies. B*73:01 exon5 was first mixed with fragmented genomic DNA with a known genotype (HLA-A*01:01:01). Next, the mixture was constructed to an NGS library and captured by STC chip according to the method described above. After enrichment, RT-PCR was performed.

### Real-time PCR

The RT-PCR were performed using the iTaq Universal SYBR Green Supermix (Bio-Rad Laboratories) on the Bio-Rad CFX96 real-time PCR detection system (Bio-Rad) with the following primers:

***GAPDH:***

forward, 5’-GCTGAGTACGTCGTGGAGTC-3′; reverse, 5’-GGCTGTTGTCATACTTCTCATGG-3′; 247-bp product.

***HLA-A*** **outside-probe:**

forward, 5’-TAATACCTCATGTGGGTCTGCCT-3′; reverse, 5’-CTAGTGCTCATGCACTGCCTG-3′; 135-bp product.

***HLA-A*** **inside-probe:**

forward, 5’-GGGTCTCAGCCACTCCTCGTCCCC-3′; reverse, 5’-GCCTCGCTCTGGTTGTAGTAG-3′; 294-bp product.

***HLA-B*** **outside-probe:**

forward, 5’-TGGGACTGCATGGAGCACTC-3′; reverse, 5’-CCAGACTGTGGATCTGTAACTCTG-3′; 183-bp product.

***HLA-B*** **inside-probe:**

forward, 5’-GGTCGGGCGGGTCTCAGCC-3′; reverse, 5’-TGGGCCTTGTAGATCTGTGTGTT-3′; 242-bp product.

***HLA-C*** **outside-probe:**

forward, 5’-AAGCAGTAGAAGAGCCTGGCA-3′; reverse, 5’-ATGCAGTCCCAATGCTCTTCA-3′; 215-bp product.

***HLA-C*** **inside-probe:**

forward, 5’-CGGGCGGGTCTCAGCCCCTCCTCGC-3′; reverse, 5’-CCTGGCGCTTGTACTTCTGTGTCTC-3′; 241-bp product.

HLA-A*01:01:01 **exon5 primer:**

forward, 5’-AGCTGTCTTCCCAGCCCA-3′; reverse, 5’-GACCACAGCTCCAGTGATC-3′; 86-bp product.

HLA-B*73:01 **exon5 primer:**

forward, 5′- AGCTGTCTTCCCAGCCCACCATCCC-3′; reverse, 5′- GCAGCGACCACAGCTCCAGTGATC-3′; 91-bp product.

### Cost measurement of STC-Seq and other common NGS HLA typing approaches

The capture chip cost of STC-Seq is 0.5 USD/sample. The NGS library cost is 10 USD per sample (en.vazyme.com). So the cost of STC-Seq is around 16 USD per sample.

Capture probe pool used by HLAssign is from Agilent. Agilent custom SureSelect (Cat NO. 5190–4859) provides 55 k oligos which can cover every base-pair of the CDS of 14 HLA genes. The list price is 124,589 USD for 480 samples (259 USD/sample). The cost of NGS library is 10 USD/sample.

Illumina Trusight HLA V2 (Cat NO.20000215) is an amplicon-based NGS HLA typing kit which costs 5000 USD for 11 HLA genes of 24 samples (208$/sample).

The sequencing cost is around 5 USD (0.3 M, pair-end 100 bp on Illumina X10 platform) per sample.

### HLA allele-calling pipeline

1. **Removing alleles with insufficient coverage:**
2. We first generate all possible artificial reads (70 bp) using the large exons (> = 70 bp) of all of the alleles of the 14 HLA genes from the IMGT/HLA database.
3. After removing PCR duplications, we converted the sequencing reads to FASTA format and used them as a mapping reference.
4. We aligned the artificial reads against the mapping reference using bowtie (bowtie -S -k 1 --best -p 20 --solexa-quals -v 0).
5. We removed the allele(s) for which the distance of any adjacent mapped artificial reads was more than 70 bp in an exon.
6. **Initial screening:**
7. The sequencing reads (after removing PCR duplicates) were mapped to the coding regions of the remaining HLA alleles using bowtie (bowtie -S -a -p 20 --solexa-quals -v 0).
8. We used the remaining alleles of the same genes to build matrices in which the columns are the allele names and rows are the base positions of the longest allele of the gene. The position of a base was filled with “1” if there were mapped reads (only the position of the first base of a mapped read was counted); otherwise, the position was filled with “0”. The null position of any allele was marked with “1” if the corresponding position of any other allele of same gene had a mapped read.
9. We used a window (22 bp by default) to scan the coding region of every allele and count the number of mapped reads (100 bp continuously mapped) in each window.
10. In the same matrix, we removed the allele(s) for which there was a window without mapped reads (100 bp continuously mapped) if any other allele had mapped reads.
11. **Reciprocative screening:**
12. The deduplicated reads from step 1 were aligned against the large exons (> = 30 bp) of the remaining alleles (bowtie2 --local -a -p 15 -N 0 --rdg 200,199 --mp 200,199 --np 100). For exons (< 70 bp), only sequencing reads with a continuously aligned length equal to the length of the exon were regarded as mapped reads. For exons (> = 70 bp), only sequencing reads with continuously aligned length reads > = 70 bp were regarded as mapped reads.
13. We pooled the alleles with > = 6 unique mapped reads (only mapped to one allele of the 14 + H&Y HLA genes) and considered them to be the “real allele pool” of the 14 + H&Y HLA genes. (We added the alleles of H and Y genes at this step because they are highly similar to the A gene.)
14. We randomly paired the remaining alleles for the same gene. The reads that specifically mapped to one allele in a pair were regarded as allele-specific reads (ASR).
15. If one allele had 15-fold fewer ASRs than another allele, or if one ASR was 0 but another was non-zero, the allele was marked as a potential false allele.
16. We excluded the potential false alleles.
17. We randomly paired the remaining alleles for the same gene again. If the reads from an allele pair and alleles of the other 13 + H&Y genes from the real allele pool could explain the ASRs of any allele in any pair for the same gene (maximum of 8 unexplainable ASRs), we added one point to the pair.
18. We added the allele pair(s) of the gene with the highest score to the real allele pool.
19. Step 14 was repeated with the updated real allele pool from step 15.
20. We retained the allele pair(s) with the highest scores for each gene.
21. If only one of the ASR(s) in the pair of remaining A genes could be completely explained by all of the remaining allele(s) of the H and Y genes, we removed this allele. If only one of the ASR(s) in a pair of remaining C genes could be completely explained by all of the remaining allele(s) of the B gene, we removed this allele.
22. The remaining allele(s) were finally reported as true allele(s).
23. The analysis pipeline also performs a quality checking for the results of every gene (PASS or NOT PASS): The analysis pipeline calculates the value of the highest score from step 17 divided by the number of all possible allele pair (s) from step16. If the value = 2, the allele pair was marked as PASS (FDR = 0.028; from 1064 loci). If the value < 2, the allele pair was marked as NOT PASS (FDR = 0.66; from 67 loci). The user should consider incorrect alleles or rare novel alleles and confirm them using another approach, for example, the Sanger sequencing or the third generation sequencing in NOT PASS cases.
24. **Null-allele checking:**
25. If the reported alleles include HLA-A*01:01:01, HLA-A*03:01:01, HLA-A*16:01:01, HLA-A*29:01:01, HLA-A*31:01:01, HLA-B*15:01:01, HLA-C*15:02:01, HLA-DRB1*04:01:01 or HLA-DRB4*01:03:01, the analysis pipeline will check how many non-PCR-duplicated reads supporting the genotype of corresponding null alleles (Additional file 5: Table S2). The pipeline will report a null allele if there are more than three non-PCR-duplicated supporting reads.

The code of STC-Seq is available for non-commercial use only:

http://bigd.big.ac.cn/biocode/tools/BT007068.

### Typing by HLAssign pipeline

We ran HLAssign on our data (each 0.25 M) with its default parameters.

## Additional files

Additional file 1: Figure S1. The length distribution of double-stranded baits is from 200 bp to 1500 bp. (TIFF 281 kb)

Additional file 2: Figure S2. Comparison of the sequence identity between the STC baits and exons (> 23 bp) of all IMGT doucumented HLA-DPA1/DPB1/DQA1/DRA/DRB3/DRB4/DRB5/E/G alleles. (TIFF 19329 kb)

Additional file 3: Figure S3. Exhibition of the exon sequences (> 23 bp) of HLA-DPA1/DPB1/DQA1/DRA/DRB3/DRB4/DRB5/E/G alleles which have the minimum sequence identity with their corresponding STC baits. (TIFF 27902 kb)

Additional file 4: Table S1. The typing results of Fig. 2c and Fig. 2d. Sheet 1–2: typing results of STC-Seq and HLAssign datasets; Sheet 3–7: typing results of HLA-A/B/C/DRB1/DQB1 at different depth. (XLSX 296 kb)

Additional file 5: Table S2. The sequences used to ID HLA null alleles. (XLSX 11 kb)

## Abbreviations

ASR: Allele-specific reads
CDS: Coding sequences
CMDP: China Marrow Donor Program
gDNA: Genomic DNA
HLA: Human leukocyte antigen
MHC: Major histocompatibility complex
NGS: Next-generation sequencing
RT-PCR: Real-time polymerase chain reaction
SBT: Sanger-sequencing-based typing
SSOP: Sequence-specific oligonucleotide probes
SSP: Sequence-specific primers
STC-Seq: Saturated Tiling Capture Sequencing
