# Supplementary figures and images for: High-sensitivity HLA typing by Saturated Tiling Capture Sequencing (STC-Seq)
Source: BMC Genomics. 2018 Jan 15;19:50. doi: 10.1186/s12864-018-4431-5 (PMC5769328; doi:10.1186/s12864-018-4431-5)

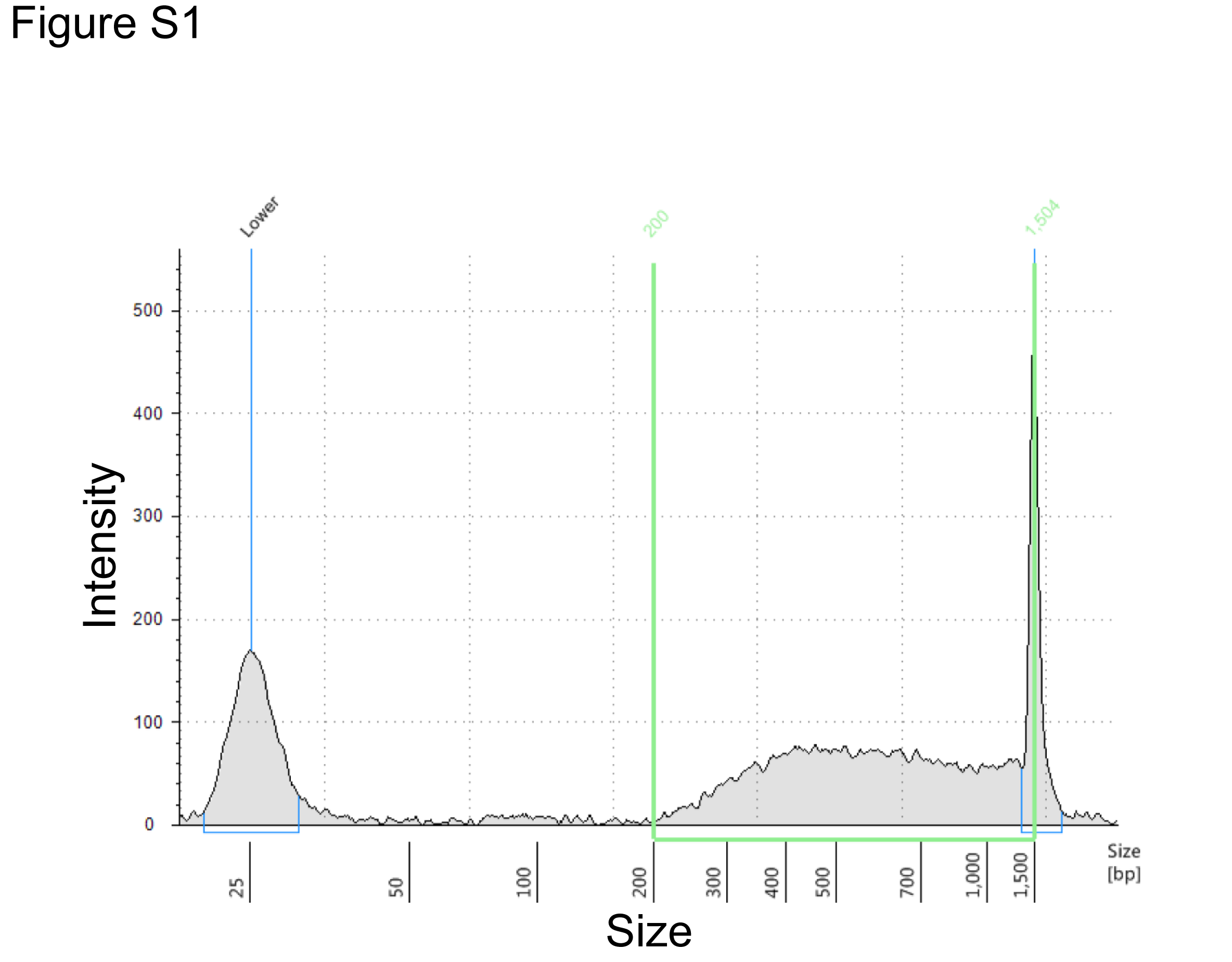

Supplement: Supplementary file 1 — The length distribution of double-stranded baits is from 200 bp to 1500 bp. (TIFF 281 kb) [file 12864_2018_4431_MOESM1_ESM.tif]

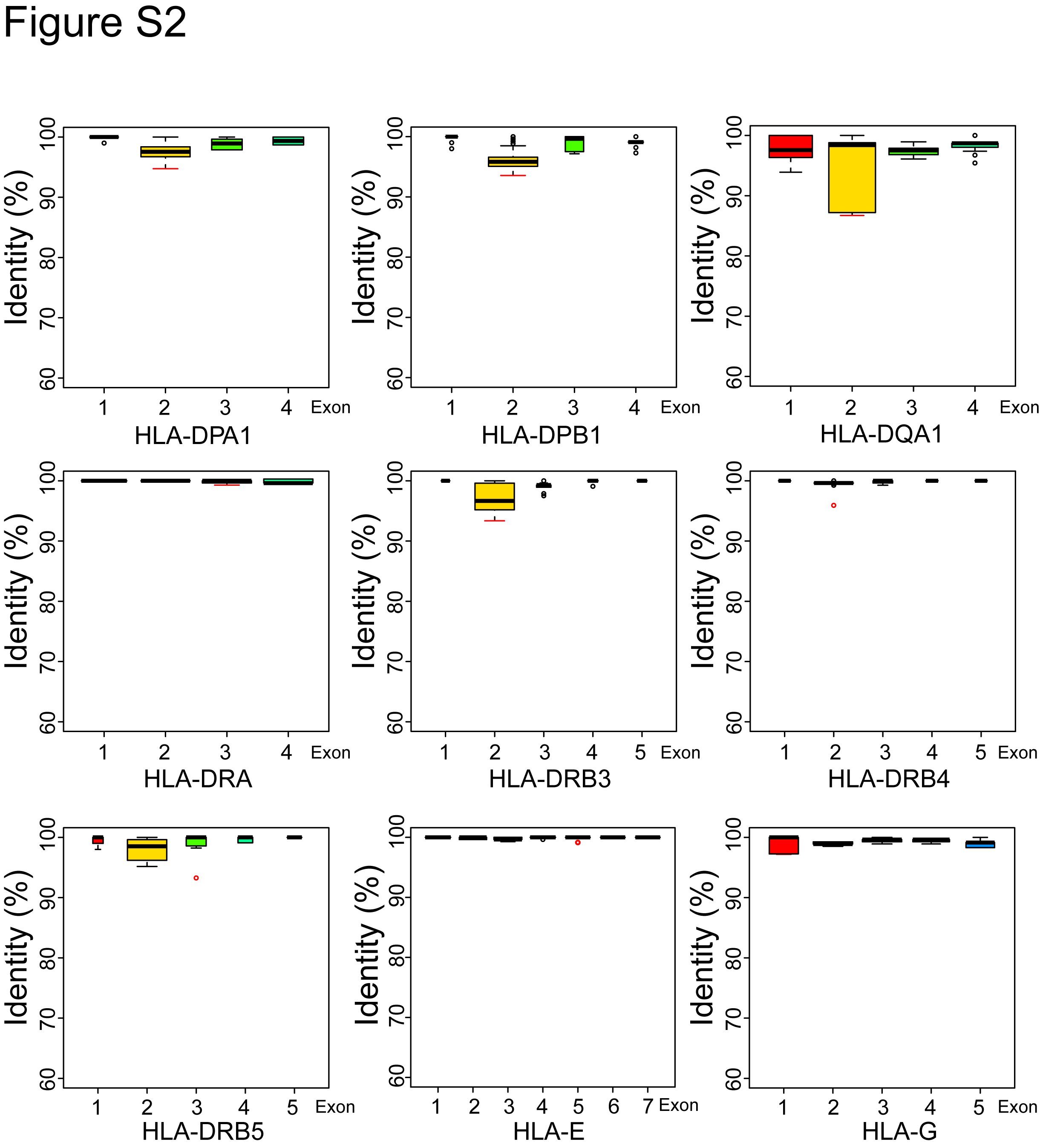

Supplement: Supplementary file 2 — Comparison of the sequence identity between the STC baits and exons (> 23 bp) of all IMGT doucumented HLA-DPA1/DPB1/DQA1/DRA/DRB3/DRB4/DRB5/E/G alleles. (TIFF 19329 kb) [file 12864_2018_4431_MOESM2_ESM.tif]

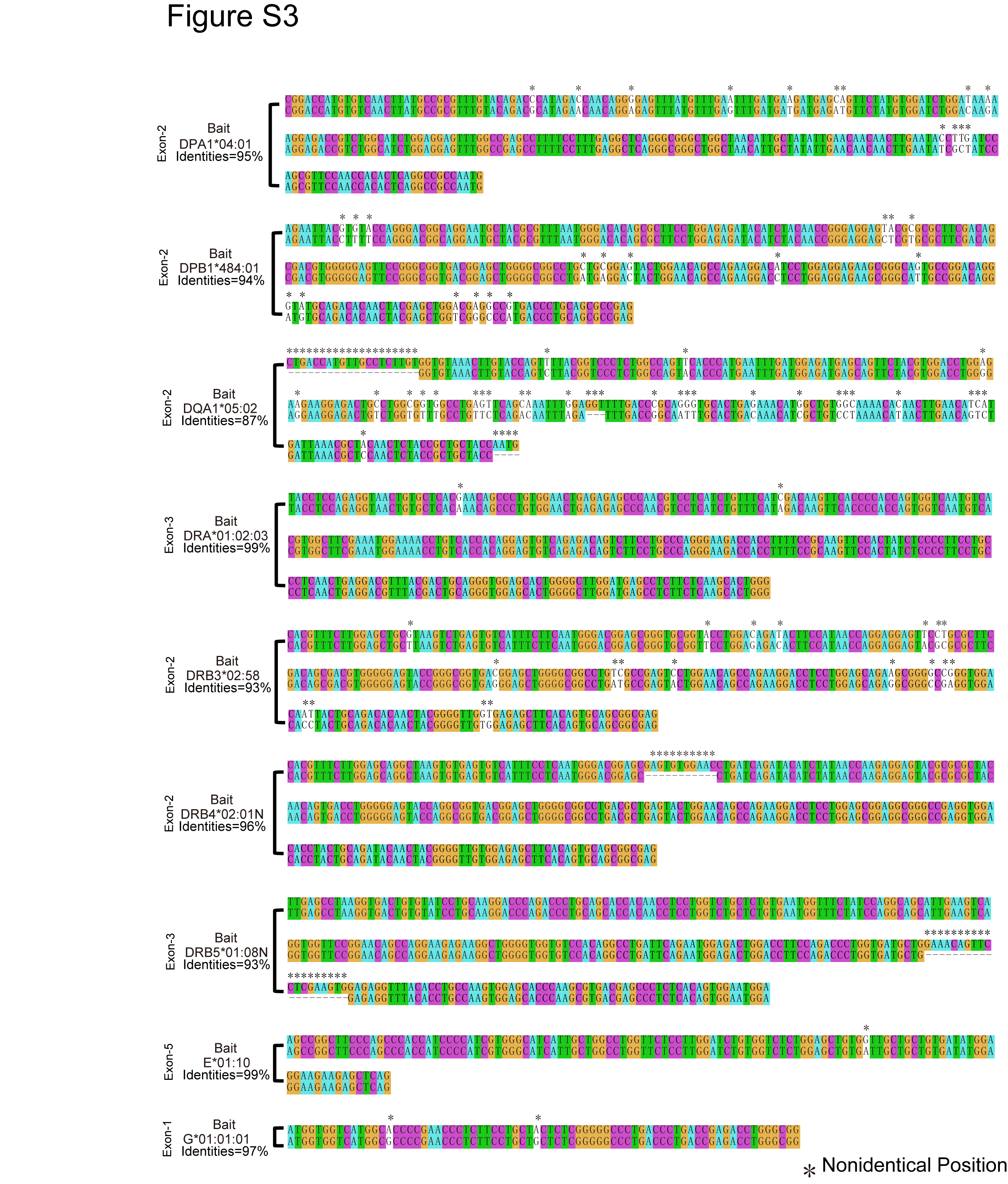

Supplement: Supplementary file 3 — Exhibition of the exon sequences (> 23 bp) of HLA-DPA1/DPB1/DQA1/DRA/DRB3/DRB4/DRB5/E/G alleles which have the minimum sequence identity with their corresponding STC baits. (TIFF 27902 kb) [file 12864_2018_4431_MOESM3_ESM.tif]
